# Supplementary material for: The In Vivo Net Energy Content of Resistant Starch and Its Effect on Macronutrient Oxidation in Healthy Adults
Source: Nutrients. 2019 Oct 16;11(10):2484. doi: 10.3390/nu11102484 (PMC6835355; doi:10.3390/nu11102484)
Supplement: Supplementary file 1 [file nutrients-11-02484-s001.pdf]

nutrients

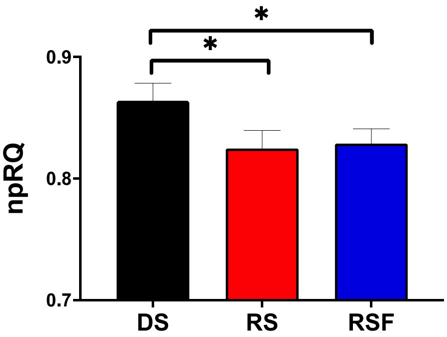

Supplementary Figure 1. Non-protein Respiratory Quotient (npRQ). 24 h npRQ, following consumption of digestible starch (DS), resistant starch (RS), or resistant starch with high fiber (RSF), as measured by whole-room indirect calorimetry. Data are expressed mean ± SEM; * $p < 0.05$.
